# Teaching Group Yoga-For-Wellness Classes via Video-Based Telehealth: Perspectives From Veterans Health Administration Yoga Teachers

**DOI:** 10.1177/27536130251382175

**Published:** 2025-10-08

**Authors:** Tony O. Pomales, Mary K. Good, Molly Delzio, Rashmi Mullur, Francesca M. Nicosia

**Affiliations:** 1 VA Office of Rural Health, Veterans Rural Health Resource Center – Iowa City (VRHRC-IC), Iowa City Veterans Affairs Healthcare System, Iowa City, IA, USA; 2 Center for Data to Discovery and Delivery Innovation (3DI), San Francisco Veterans Affairs Healthcare System, San Francisco, CA, USA; 3 University of California, San Francisco, CA, USA; 4 Greater Los Angeles Veterans Affairs Healthcare System, Los Angeles, CA, USA; 5 University of California, Los Angeles, CA, USA

**Keywords:** yoga teachers, yoga, telehealth, veterans, qualitative methods

## Abstract

**Background:**

Online yoga offers significant potential for healthcare systems. As efforts to incorporate online yoga in healthcare settings have grown, research in this area has mostly focused on the perspectives of patients and clinicians. Although yoga teachers are crucial to the implementation and delivery of telehealth yoga (tele-yoga), their views on this topic have not yet been adequately considered. This study examined the perspectives of Veterans Health Administration (VA) yoga teachers regarding a VA Office of Rural Health-funded multi-site tele-yoga implementation study.

**Objectives:**

This qualitative study aims to explore VA yoga teachers’ perspectives and experiences with regards to implementing and delivering synchronous, remote, group yoga-for-wellness classes via video-based telehealth and provide lessons learned.

**Methods:**

A qualitative study was conducted with 13 yoga teachers from 6 different VA healthcare systems included in a multi-site tele-yoga implementation study. Interviews covered experience with teaching in an online format, program implementation and delivery, and equity considerations. Interviews were conducted and recorded using Microsoft Teams. Transcripts were coded using MAXQDA 2022 and analyzed via thematic content analysis.

**Results:**

VA tele-yoga teachers identified 4 categories impacting tele-yoga implementation and delivery: (1) equipment and information technology needs; (2) physical space (particularly for teachers teaching onsite in a hospital or clinic setting); (3) accessibility and safety; and (4) administrative responsibilities and end-user support. Findings highlight key considerations and potential solutions for teachers and healthcare settings, and resources needed to provide a safe, accessible, and effective experience for participants.

**Conclusion:**

Learning from tele-yoga teachers’ experiences at VA can inform best practices for teaching yoga to groups in a synchronous, remote, online format within healthcare settings. The ways in which VA tele-yoga teachers surmounted barriers and harnessed opportunities for success can inform program recommendations as tele-yoga programs expand across VA and become incorporated in other healthcare systems and facilities.

## Introduction

Efforts to implement yoga programming in healthcare settings in the United States have increased over the last 2 decades as the evidence base for an integrated approach to treating certain clinical conditions (eg, PTSD, chronic lower back pain) and improving general wellbeing has grown.^1-7^ In the U.S. Veterans Health Administration (VA), internal policy has supported the implementation and expansion of yoga programs as part of a Whole Health System of care that combines conventional medical treatment with complementary and integrative health (CIH).^8-12^ As an evidence-based CIH approach, yoga may be included in a veteran’s medical benefits package when viewed as clinically necessary, or when accessed as part of Whole Health (WH) programs to support general wellbeing.^12^

VA now offers yoga nationwide with options for sessions in-person and via telehealth.^11^ Significant investments in telehealth technology in the mid-2010s expanded access to yoga through video-based telehealth platforms like VA Video Connect (VVC) – VA’s secure, real-time, interactive videoconferencing system that links patients at home to a provider at another location – and similar other platforms approved by VA.^13^ Over the last decade, video-based telehealth yoga (tele-yoga) has been successfully implemented across many VA healthcare systems, benefitting veterans in different geographical locations while reducing barriers to participation for rural veterans, veterans who face travel challenges, and veterans with limited mobility and other health challenges.^13-19^

During the COVID-19 pandemic, VA expanded telehealth delivery of CIH and Whole Health services, modifying services as needed for successful delivery.^13,16,20,21^ Although telehealth had been used pre-pandemic to improve veteran access to CIH modalities, the need to widely and quickly implement movement-based CIH modalities on a large scale created both new opportunities for expanding access to yoga and also new challenges for yoga teachers with little to no experience teaching movement-based classes to groups in a virtual setting.^13,16,20,21^

Over the last several years, the growing demand for tele-yoga has been accompanied by research examining its acceptability and perceived effectiveness, largely assessed through the perspectives of patients and clinicians.^15-17,22-25^ While this research has supported continued investment in yoga delivered through telehealth, limited information is available about the realities, practicalities, and potential constraints of implementing and delivering tele-yoga in real-world healthcare settings, especially in a large, integrated healthcare system like VA. We suggest that in order to understand contextual factors that may contribute to successful implementation and delivery of tele-yoga in health care settings, greater attention to the perspectives of instructors teaching in these settings is needed. Using a qualitative descriptive design, we analyzed interviews with VA yoga teachers to describe their (1) perceived concerns, priorities, and perspectives regarding tele-yoga implementation and delivery, (2) experiences with teaching yoga online in a healthcare setting to patients, (3) adaptations to evolving challenges, and (4) considerations and potential solutions for ensuring patient safety and accessibility and improved delivery of tele-yoga to heterogeneous groups of patients with wide ranges of ages, mobility, and health conditions.

### Multi-Site Tele-Yoga Implementation Study

The VA is the largest integrated healthcare system in the United States, serving over 9.1 million enrolled veterans each year. According to the VA Office of Rural Health (ORH), of the 4.4 million veterans who live in rural areas, 2.7 million are enrolled in VA health care. Rural-dwelling veterans tend to be older, have lower income, and are more medically complex than their urban counterparts, requiring significant ongoing access to medical care.^26^ ORH funds programs that use telehealth to improve access to care for veterans living in rural areas. In fiscal year 2021, ORH provided funding for a pilot tele-yoga program based at the San Francisco VA Healthcare System.^16^ Based on the success of this pilot, the program was expanded with subsequent ORH funding to 6 additional VA healthcare systems (also referred to as “sites” in this paper) across 5 Veterans Integrated Services Networks (VISNs).^16^ Sites represented a mix of urban medical centers and associated community-based outpatient clinics serving primarily rural populations (see Table 1). Funding was provided to each site for yoga teacher salary support, audio-visual (AV) equipment (either as a new addition or as updates to existing equipment provided by the local healthcare facility), and yoga supplies (eg, mats, blocks) for teachers and participating veterans. The 6 sites were given autonomy over aspects of their local tele-yoga program including management, types of AV equipment they used, hosting platform (eg, VVC), and other elements to take into account the variation in pre-existing programs and institutional organization. All sites were provided with implementation guidance and facilitation throughout the process, as described below.

**Table 1.** Tele-Yoga Program Site Characteristics

Characteristic	Site 1	Site 2	Site 3	Site 4	Site 5	Site 6
Tele-yoga offerings prior to joining multi-site tele-yoga implementation study	Yes	Yes	No	No	No	Yes, through the Veterans Yoga Project (VYP)
Telehealth platform used	VVC	VVC	VVC	Webex	VVC	VVC, Zoom (VYP)
Service line(s)	Integrative Health and Primary Care	Physical Medicine & Rehabilitation, then Whole Health	Recreational Therapy and Whole Health	Whole Health	Physical Therapy and Whole Health	Recreational Therapy and Whole Health
Admin support staff	No	Yes	No	Yes	No	No
Yoga props offered to participants	Yes	No	Yes	Yes	Yes	Yes
Total number of participating patients (N)	244	90	100	86	292	56
Rural-dwelling participants (%)	2	29	29	35	11	7
Female (%)	39	51	31	42	43	38
Age (% 65+)	37	22	46	20	18	48
Race (%)						
White	45	46	88	47	55	34
Black/African American	26	26	4	10	27	52
Asian	7	10	No data	15	1	No data
Native Hawaiian/Pacific Islander	2	4	No data	12	0	2
American Indian/Alaskan Native	1	3	No data	1	1	2
Ethnicity (%)						
Hispanic or Latino	12	9	2	13	5	21

Classes were delivered to groups of veterans remotely through VVC or another VA-supported telehealth platform permitted during the pandemic (eg, Webex) (see Table 1). Use of VVC or another approved hosting platform was determined by leadership at each site with the goal of protecting patient privacy and information. Veterans were referred for group tele-yoga classes by various clinical staff (eg, primary care providers, mental health providers, physical therapists, social workers, and Whole Health coaches). Some sites mandated veterans to receive clearance from a medical provider before enrolling in tele-yoga. Veterans received a secure weblink to log into the video-based classes. At most sites, yoga classes were taught by VA employees with healthcare backgrounds who were also certified yoga teachers with a minimum of 200 h of training. Classes across sites typically lasted 1 h. Sites offered a minimum of one class per week per teacher (range 1-4 classes per week per teacher depending on teachers’ FTE, or full-time equivalent, in the program), and were encouraged to include at minimum one chair-based group class per week to ensure access for veterans with limited mobility and other health challenges.

Program sites had autonomy in designing their tele-yoga programs while receiving support from the study implementation facilitation team led by the study’s principal investigator, an experienced health services researcher and certified yoga therapist. Implementation facilitation meetings occurred via Microsoft (MS) Teams on a monthly or bimonthly basis from the Fall of 2021 to the Spring of 2023. Program sites joined the implementation study at different stages with their tele-yoga programming. As previously mentioned, the COVID-19 pandemic led to a rapid expansion of telehealth services across VA, including tele-yoga. The pandemic significantly shaped the implementation of tele-yoga at all program sites and continued to affect sites for most of the time this study was taking place. In fact, some sites were already offering tele-yoga to groups prior to joining the study – having rapidly moved this service online with the onset of COVID-19 – while others were in the process of switching their group classes to an online format (see Table 1). One site did not have an existing yoga program prior to joining the study (see Table 1). Implementation facilitators provided guidance on hiring and credentialing yoga teachers, IT and AV equipment, yoga supplies, and processes to increase access and reach more geographically isolated veterans.

## Methods

### Study Design

We conducted an evaluation of tele-yoga program implementation guided by the Reach, Effectiveness, Adoption, Implementation, and Maintenance (RE-AIM) framework.^27,28^ RE-AIM is commonly used in program evaluations, providing a framework to assess factors impacting program adoption, participants reached, implementation outcomes, and program effectiveness. Given the goal of reducing barriers to access due to geographic location and other social determinants of health, we also incorporated elements of the Health Equity Implementation Framework (HEIF) in our evaluation.^29,30^ HEIF includes additional constructs affecting implementation and helps to assess health equity determinants. This study was part of a larger quality improvement initiative focused on barriers and facilitators to program implementation and delivery, and veterans’ and yoga teachers’ experiences with the program. The San Francisco VA Healthcare System Human Subjects Protection Program determined the activities of this quality improvement project to be non-research and exempt from institutional review board (IRB) approval.

### Data Collection

To understand factors affecting tele-yoga implementation and delivery, we invited all tele-yoga teachers from the 6 program sites to participate in individual, semi-structured interviews. After obtaining verbal consent, interviews were conducted and recorded by a qualitative researcher on the team (TP) using MS Teams. Auto-generated transcripts were reformatted, edited, and validated by project team members (TP, MKG, MD) using the audio-recording for comparison. Yoga instructors were interviewed on a rolling basis between February 2022 and June 2023 as new instructors were onboarded. Interview questions covered instructors’ motivations for teaching yoga to Veterans, how they became involved with their site’s tele-yoga program, challenges they faced while implementing and delivering virtual yoga classes to groups of Veterans, adaptations for teaching yoga virtually in a healthcare setting, and recommendations for ongoing program improvement.

### Data Analysis

Validated interview transcripts were loaded into MAXQDA 2022 qualitative research software for coding and analysis (VERBI Software. *MAXQDA 2022*. Berlin: VERBI Software, 2021). The coding framework was developed by the 2 qualitative researchers (TP, MKG) on the team with guidance by the PI (FMN) and included deductive codes derived from RE-AIM and HEIF as well as inductive codes that were identified during close readings of the transcripts. Codes were cross-checked for reliability and consistency across researchers by simultaneous coding of 2 transcripts at the initiation of coding, then frequent check-ins between researchers throughout the coding process. Analysis followed a thematic content analysis approach.^31^

## Results

### Participant Characteristics

Thirteen yoga teachers from six program sites completed an individual interview. Eleven (85%) were women and two (15%) were men. The two men were veterans (15%), and the eleven women were non-veterans (85%). Most yoga teachers were dually licensed, holding various clinical positions in VA. Recreational Therapist (n = 4), Physical Therapy Assistant (n = 3), and Whole Health Coach (n = 3) were the most common clinical positions, followed by Physical Therapist (n = 1), Social Worker (n = 1), and Dietician (n = 1). One yoga teacher was hired as a contractor. Ten (77%) had been employed at the VA for three or more years, reporting three or more years of yoga teaching experience at VA. Most (85%, n = 11) reported having no previous experience teaching yoga in an online format prior to the pandemic. Two-hundred hours of training was most frequently reported (77%, n = 10), followed by 500 h and yoga therapist certification (23%, n = 3 each). The most common styles of training reported were general Hatha yoga (69%, n = 9) and vinyasa flow (31%, n = 4). Eight teachers (62%) reported having veteran-specific yoga teaching certifications that included an internal VA-supported, yoga teacher training program and community-based yoga training programs focused on veterans.

### Interview Findings

Yoga teachers’ perceived concerns, priorities, and perspectives regarding tele-yoga implementation and delivery were grouped into 4 categories: (1) equipment and information technology (IT) needs; (2) physical space; (3) accessibility and safety; and (4) administrative responsibilities and end-user support. A summary of the 4 categories with specific considerations and potential solutions is presented in Table 2.

**Table 2.** Key Considerations for Tele-Yoga Implementation and Delivery

Category	Key considerations	Potential solutions
1. Equipment and IT needs	• Reliable internet connection • Stable telehealth platform connection • Audio and visual (AV) quality • IT support for teachers • Suitability of VA Video Connect (VVC) for large, movement-based classes • Yoga supplies for participants	• Effective, quality AV equipment (eg, Bluetooth headsets, large monitors, high-resolution webcams) • Adapt VVC to accommodate larger groups • IT personnel dedicated to virtual CIH classes • IT training and additional technical support for teachers • Providing participants with yoga supplies; providing creative home solutions when supplies are not available for participants
2. Physical space	• Adequate physical space, esp. when teaching onsite at a hospital or clinic – clean, well-lit, adequately sized for movement, demonstration, and prop use • Workflow – going from teaching yoga to other clinical responsibilities (timing, clothing changes)	• Dedicated room onsite for virtual CIH classes • Flexibility to telework and teach from home • Build in additional time for provider/staff-trained yoga teachers to change and go from teaching to their other clinical responsibilities
3. Accessibility and safety	• Teaching to heterogenous groups with wide range of ages, mobility, health conditions • Trauma-affected participants • Pain-affected participants	• Training to teach clinical and medical complex populations • Train in/default to using trauma-informed language • Be able and flexible enough to make changes to practice based on attendance • Provide different verbal cues to different participants that are clear and easy to follow • Awareness of safety procedures for falls or other medical emergencies • Medical clearance for movement-based classes • Moving gently through different poses, esp. from floor to standing and vice-versa • Learn as much as possible about participants to tailor sequences and provide modifications in real time • Checking in with participants before and after class • Prioritizing chair-based yoga • De-emphasize physically intense movement in favor of proprioception and body awareness
4. Administrative responsibilities and end-user support	• Increased workload • Dual responsibilities of teaching and administering yoga program (scheduling classes, emailing weblinks, teaching, orienting participants, managing yoga supplies, documenting participation, responding to participant concerns, emails, etc.)	• Full-time or part-time medical/program support assistant for yoga programs • Additional technical support for end-users/participants provided by IT specialists
	• Teachers may have other clinical responsibilities/roles • Technical support for end-users/participants before and during class	

#### Category 1. Equipment and Information Technology (IT) Needs

VA yoga teachers highlighted the importance of reliable internet connectivity, audio and visual quality, and IT support for effective and safe delivery of tele-yoga for groups. While most healthcare workers rely on a strong internet connection for patient care, high-speed internet is essential for delivering online health resources like tele-yoga. A weak connection not only compromises the video and audio quality needed for a virtual yoga class but can also lead to dropped calls, which are not easily recovered in a healthcare system, because of the need to ensure secure communication to protect patient data. For example, Yoga Teacher 2 reported experiencing “technology failures quite frequently,” more often at the start of the pandemic when CIH offerings like yoga were being transitioned to online delivery. “[T]he internet just drops or something in the middle [of class]. I don’t know what happens, but [the participants] can’t see me or hear me. And then, I have to sign all the way out and go back in and it’s pretty disruptive” (Yoga Teacher 2). Because VA telehealth requires veterans to sign in using a secure weblink, teachers reported challenges resuming class after a disconnection or internet disruption: “It would take up so much of the time…just trying to get people signed on” (Yoga Teacher 2).

While most yoga teachers found the opportunity to expand the reach of yoga for wellness highly motivational, they were concerned about the effect that lacking certain audio-visual (AV) equipment (eg, Bluetooth headsets, large monitors, and high-resolution webcams) and yoga supplies (eg, mats or blocks for veterans to use at home) could have on certain groups of veterans with different physical capacities and clinical conditions. For example, a majority of the yoga teachers interviewed reported that at the start of the transition to virtual yoga (that is, prior to joining the tele-yoga implementation study) their AV equipment was either outdated or inadequate, with a small number of teachers noting that they lacked basic AV equipment (eg, a laptop or tablet) entirely. One teacher described how she “teach[es] from the iPad for the most part, but there’s issues with that…The camera is not very good.” (Yoga Teacher 3). Another mentioned how “even though I use a pretty good microphone, and the sound should be pretty good, it’s just hard for [the participants] to hear” (Yoga Teacher 2). Teachers who only had access to a device with a small screen (eg, a laptop or iPad) described how this impacted delivery and their ability to see all veteran participants:

“When we very first started, I was using my laptop on the floor…and it was very hard to see the patients, but still, they could see me and they could see what I was doing, and I was giving lots of verbal instructions…But I mean, you definitely want to be able to see them as well…that's a very important factor…so you can make corrections, so you can give them suggestions…I really just prefer being able to see what they're doing…For safety too! I actually had a patient fall one time in my class, and I didn't even know he fell. So, I think it's extremely important to be able to see your patients.” (Yoga Teacher 7).

Yoga teachers persevered with tele-yoga classes in spite of equipment-related challenges, adapting their instruction by taking pains to provide very clear verbal cues and slowing down the transitions between movements or postures to ensure participation and safety. Teachers continued with these practices even after obtaining improved AV equipment.

The suitability of VVC for delivery of group-based yoga was yet another concern raised by teachers. Because VVC was originally designed for one-on-one consultations between one provider and one patient, a difficulty that teachers collectively faced was the inability to see all participants on their screen when groups were larger than 8 people. As one teacher explained, “So, like on [MS] Teams…we can see each other really well. You’re big, I’m small in the corner, everything else like that. Whereas on VVC, if I want to be able to see everybody in the same size, you know, 2-inch, 3-inch square, that’s what they’d be seeing, too. So that’s a big hindrance” (Yoga Teacher 4). The inability of participants to see the instructor in “speaker view” was viewed by yoga teachers as a potential barrier to participation and effectiveness. Teachers reported that it prevented them from being able to provide more individualized, tailored instruction or to see when adjustments to yoga poses were needed (eg, if most participants were struggling with a particular pose because of limited mobility). Because decisions about which telehealth platform to use for tele-yoga were made by site leadership – with a strong preference towards VVC to ensure secure communication and protect patient data from breaches – teachers recommended that VVC be adapted to accommodate larger groups.

Suggestions for improved IT support were also common. While some teachers stated that the IT support in their local facility was robust, several others found their respective VA facilities lacking in this area. Many expressed being intimidated or frustrated when IT problems arose during class. For example, one teacher told us, “[Having] someone that can be available to us while we’re on the VVC platform, that can really help with the tech problems, ‘cause I’m not -- I don’t know how to do half this stuff.” (Yoga Teacher 3). Teachers also voiced a need for training in the more technical aspects of virtual yoga instruction to groups. Although a couple of teachers had experience using VVC to teach yoga in a one-to-one format, most pivoted to teaching yoga classes to groups online somewhat hastily at the start of the pandemic and thus had to learn “on the fly.” As one teacher recalled: “In the beginning, it was hard. It was really hard, because, in the heart of COVID, we had to kind of figure all this new technology out at the drop of a dime, and it was difficult because I didn’t know how to make a group [in VVC]” (Yoga Teacher 3).

In addition to IT support and AV equipment needs, teachers also discussed the need for yoga supplies (ie, mats, blocks, bolsters, straps, chairs) for participants to use at home. Although most teachers did not believe that a lack of yoga supplies posed a major barrier to participation, some felt the supplies could enhance the experience for participants and expressed how not having access to yoga supplies could lead some participants to lose interest in tele-yoga. For example, one teacher said, “[T]hat’s a big part of yoga…to have even a chair, a good chair…like a dining room chair. Some people don’t even have that, so it’s been challenging to keep them on board and keep them coming” (Yoga Teacher 13). Several teachers mentioned “home solutions” they recommended to veterans in the place of standard yoga props, such as encouraging participants to “practice on a towel, if that’s what they had…or we might have started with a chair, just because we didn’t know if people had mats” (Yoga Teacher 10). However, during her interview, Yoga Teacher 12 noted that “home solutions” for yoga props may require advanced planning. As she explained, “you can adapt…you can get creative. But it’s not as easy for some people to adapt in the moment…I will often say, ‘If you don’t have a yoga strap, then you can use the tie of a robe, or a necktie, a TheraBand even, a sweatshirt, towel, really anything that you can link onto that area of the body.’ But, sometimes, I think, if they had that yoga strap right there in front of them that would be easier for them to just access” (Yoga Teacher 12). Most sites in the tele-yoga implementation study eventually went on to offer yoga props to participants (see Table 1), motivated in part by the concerns teachers expressed at implementation facilitation meetings.

#### Category 2. Physical Space

Availability of physical space for effective tele-yoga delivery was a unique challenge faced by most of the teachers we interviewed who taught onsite from a VA healthcare facility. All the teachers we interviewed agreed that an *adequate* physical space was a critical necessity. Most teachers regularly taught onsite in a hospital or clinic and described how “space constrictions” posed significant obstacles to effective delivery of tele-yoga. Because a clean, well-lit, and adequately sized room was not always available, yoga teachers often had to adapt their personal offices for tele-yoga instruction. For example, one teacher told us: “…it’s like a full-on industrial office. It’s not the most inviting setting…It’s loud, and it’s dirty…When I come in here, I start off the day mopping the floors [in my office], because I know I’m gonna put my mats down on it…Space is such an issue [in our facility]” (Yoga Teacher 9). One teacher, who shared an office with her colleagues, explained how her solution was to adapt a small, multi-purpose conference room by using a mobile equipment cart.

“I adapted [the conference room] to be an exercise [room]. The computer and the laptop are attached to a cart that is ours for [department-related activities], and it travels with us when we get ‘evicted’ [i.e., when she needs to vacate the conference room for the next activity or meeting to take place]. We have a large TV…But it can't be locked up. The equipment has to be wheeled back to the cabinet [in my office], because the [conference] room is not lockable and it’s open. There are veterans [who] hang out there. Sometimes, I have to tell them, ‘I need the room for an hour.’” (Yoga Teacher 8).

Given the AV equipment and security/privacy considerations associated with tele-yoga at VA, some teachers stressed the need for a dedicated room for yoga and other movement-based modalities. In fact, one teacher described how “it would be nice if we actually had our own little studio classroom where we are teaching different days, set up just for yoga or Tai Chi. When we walk in there, the cameras are already in place. That would be a wonderful opportunity, ‘cause all of the gear could be in there” (Yoga Teacher 5).

In contrast to those who were required to teach onsite, some teachers who could tele-work were able to teach tele-yoga from their homes. One teacher said, “I have equipment at home, and I have equipment here [onsite]. So, I bring my [VA-issued] laptop home with me, and I just use the camera on the laptop and the microphone on the laptop” (Yoga Teacher 10). Another, citing space limitations, said, “[P]art of the reason I’m [teaching some classes from] home is because we just--we don’t have the space to accommodate all the staff, so we rotate. We have rotating days” (Yoga Teacher 9). Among those who were able to teach yoga from home, similar physical space considerations were described as when teaching onsite. These included: (1) maintaining a clutter-free background to minimize visual distractions; (2) ensuring adequate lighting, so yoga participants could easily see the teacher’s movements; and (3) having an adequate amount of space to “to have my whole body in the frame” (Yoga Teacher 1).

Notably, some teachers who taught onsite mentioned that it was difficult, at times, to find a place to quickly change their clothes before and after class; perhaps not a critical need, but an important consideration, nonetheless, for teachers with other clinical appointments and teaching onsite in a healthcare setting. As one teacher described, “The challenge, I’ll be honest with you, is the dress code, having to change clothes into yoga attire from business casual, [then] back to business casual. It’d be just nice to freshen up in between my sessions but not have to keep changing clothes in between sessions, so that I can walk around in our clinic” (Yoga Teacher 5).

#### Category 3. Accessibility and Safety

Safety and accessibility were prominent themes in our interviews with VA yoga teachers. Many described a strong personal motivation for teaching yoga to veterans that included making yoga accessible to individuals with a variety of clinical conditions and physical abilities. At the same time, teaching yoga via telehealth to heterogeneous groups of veterans with a wide range of ages, mobility, and health conditions presented certain challenges. Teachers described a high level of preparation as well as awareness of safety considerations, including procedures for handling events such as falls or other medical emergencies. Safety protocols included facility- or system-specific policies and procedures to ensure safe participation, such as clearance by a medical provider to participate in movement-based wellness activities such as yoga. One teacher described the process for veteran enrollment in her tele-yoga classes, which included an intake assessment in addition to medical clearance: “[First], they go through a Whole Health intake and for them to go through that process they have to get the clearance by their medical provider that like, ‘Okay, they’re cleared to do gentle movement.’ And then from there when they’re connected to our yoga coordinator, if they mention any types of like injuries or anything that like would be helpful for us to be mindful of, she alerts us to that before they come into our class” (Yoga Teacher 1).

In addition to local policies and procedures implemented to ensure safety, teachers also mentioned several strategies they used to address safety and enable access for veterans with lower mobility and function. For example, one teacher informed us how, at the start of the transition to tele-yoga, she preferred to start with seated yoga classes that used a chair for balance or support while seated and later included floor-based poses as she gained more confidence in the safety of tele-yoga: “I think we started with chair [yoga] -- also because we weren’t really sure about how the safety of the yoga was gonna go. So, we started with chair yoga to build our own confidence and comfort with it and then transitioned to mat” (Yoga Teacher 10). Another teacher, who taught a class that incorporated standing poses for those with greater mobility, described how she was still very conscious of moving from floor to standing and preferred to move gently through different standing poses. She also described being inclusive of participants with less mobility, incorporating the use of chairs as needed.

“The way I've designed it, we're not going up and down all the time, I don't do any mat work [on the floor]. So, we're going to do a lot more flowing loose motions, going through the warrior poses, and moving through a good handful of different standing poses. I might do a little bit of chair work as well, depending on who's in my class and what the situation is that day.” (Yoga Teacher 4)

Because classes were often heterogeneous in terms of participant mobility and clinical conditions, all the teachers we spoke with agreed that it was critical for them to learn as much as possible about their participants before each class in order to prepare unique, tailored sequences for the class with as many adaptations or modifications as needed. Teachers also mentioned checking in with participants at the start of each class to allow for contingencies and ensure accessibility.

“[E]ach class, I try to address who’s there…they’re all so very different. I always ask them in the beginning what conditions they wanna share with me. For instance, we started a new class yesterday of women and there were three that signed up. The first one came on. She said she had COVID, so she couldn’t stay on. The second one that came on was in her son’s house, just visiting him, with lots of kids, and she had not even a dining room chair and lots of conditions…so, I worked the best I could with what I had, you know? And then, the third one had a chair. She was ready to go, so I addressed her in a different way.” (Yoga Teacher 13)

Other strategies used by teachers to ensure safety and enhance accessibility during group tele-yoga classes included giving clear verbal cues combined with visual demonstrations and de-emphasizing more physically intensive movements or poses. As one teacher explained, “I would say the in-person…I maybe will step it up a little bit more as far as physical challenges, because I’m there with them and I can see them differently. When I teach virtually, I feel like I’m teaching more of, like, ‘You’re watching a video. I’m demonstrating,’ you know? So, [the veterans are] seeing the instructor practice. They’re following along. Whereas in person, I tend to walk around a bit more, demonstrate as needed.” (Yoga Teacher 12).

In addition to physical accessibility, teachers also discussed strategies they used to encourage proprioception and body awareness and to make their classes safe and accessible from a trauma-informed lens. As one teacher explained, “[In] general, I think I teach with the knowledge and awareness that I’m gonna be offering different variations and adaptations, because people come with so many different ailments or backgrounds... And so, I think even if I wasn’t teaching to the veteran population, I would try to do that because you never know what background somebody is coming in with…Not just physically, but emotionally” (Yoga Teacher 12).

Some teachers described the effort to make classes safe and accessible from a physical and emotional standpoint as empowering participants “to make choices that feel best for them and their bodies, so offering different options [and] encouraging people to find what works best for them” (Yoga Teacher 1). Others described specific steps they took during class to support participants who experience post-traumatic stress and create an environment of psychological safety. Steps included: (1) paying attention to/avoiding potentially triggering language, (2) using empathetic and supportive language, (3) centering choice and empowerment for each participant’s level of engagement, and (4) checking in with participants after class. As one teacher with participants who have experienced both combat and military sexual trauma expounded, “I try to mitigate [post-traumatic stress] by just trying not to use potential keywords, trigger words, trigger positions. I try not to -- for an example, if we’re doing a floor exercise, I don’t say, ‘Spread your legs’…[and] with the breathing, I introduce it to them and allow them to explore it and always go back to ‘what is your body telling you?,’ ‘what is your sensing inside?’” (Yoga Teacher 5).

#### Category 4. Administrative Responsibilities and End-User Support

In addition to delivering safe, accessible, and effective yoga instruction to groups via video-based telehealth, teachers also discussed how the dual responsibilities of teaching and program administration posed additional challenges that sometimes felt like another job altogether. Teachers described being strained at times by the “behind the scenes” aspects and demands of teaching and managing participants, which included simultaneously implementing, teaching, and maintaining virtual yoga group classes. As one teacher described, “I wear many, many hats here. So, for example, people either just show up [to a virtual yoga class], or what primarily happens is I’ll get a consult for yoga. So, I’ll go into CPRS. I accept the consult. I call or email [the prospective Veteran participant]. And then from there I add them to the roster. I add them to the VVC link and then it sends out that way. I’m the only one that does any of that. I do everything. There’s no person but me…I call, follow up, email, do all that.” (Yoga Teacher 3).

Teachers at some sites mentioned additional responsibilities that stretched their capacity, such as scheduling classes, teaching multiple, different classes, and following up via email or phone with veteran participants before and after class. Altogether, this created “a lot of work outside of teaching,” as one teacher explained, “Whenever people ask me, ‘I’m thinking about starting to teach classes?’ I’ll say, just so you know, it’s not just teaching classes…like, you’re your scheduler. You have to send out the invites. You have to do the notes [in CPRS]. You have to respond to all these emails and veterans constantly emailing you asking you all these questions about classes …I love doing it, but it’s a lot of work” (Yoga Teacher 7).

As with any other healthcare service at VA, participation in yoga-for-wellness groups must be clinically documented to support patient-centered care goals and register workload hours for teachers who are also employed as clinicians (eg, a nurse care manager or physical therapist assistant). Teachers reported that clinical documentation increased their workload during working hours. The time it took to document tele-yoga participation, or patient encounters, could take them beyond the number of hours or FTE associated with the tele-yoga implementation study. Another task that could take more time than expected was the process of completing what are called “group notes.” Group notes allow a clinician to document in multiple patient charts simultaneously, which ostensibly should increase efficiency. However, teachers noted that group notes did not always work as intended, leading to a greater administrative burden with little support for troubleshooting. For example, one teacher described how “…group notes can be a pain if they don’t go smoothly, and then when you’re adding in the Whole Health factors, then that takes some additional time just because the system is very slow. And then, when you’re doing group documentation, if…something [is] missed in the encounters or connecting to the wrong clinic time or the wrong appointment time, then you have to go back in and redo them or fix them…So, that is very time consuming” (Yoga Teacher 12).

Another reported unforeseen time commitment, as well as a potential cause of stress and strain on teachers, was teaching hybrid classes, which included both online and in-person participants. Hybrid classes not only required teachers to set up a room onsite (in the medical center or clinic) for in-person participants and arrange the technology needed for those attending class via telehealth, but it also required teachers to create separate group notes for the 2 types of participants. Hybrid classes, thus, often implied greater demands placed on teachers, as one teacher described: “I did not realize how much more work that would cause. I thought, ‘Oh, I’m already teaching this class online, I might as well just have people come [in].’ Didn’t even think about the fact that, ‘A,’ I’ve gotta set up the room for those patients to come, [and] ‘B,’ [in-person participants] like to stay and talk, you know? And then, you have to do 2 separate notes. You’ve got to do an online VVC note, and then you’ve got to do an in-person note, and I’m like, ‘Oh my God, this has just doubled my work’” (Yoga Teacher 7). One solution proposed by yoga teachers to lessen the burden of teaching and administrating a tele-yoga program was to hire a full-time or part-time medical or program support assistant to take over certain administrative tasks and allow teachers to handle their varied responsibilities more effectively. Some teachers implied that this support could even improve veteran access: “If we could have one administrator of some sort having more control over the classes, making those phone calls, getting the veteran signed up for it, everything else…that would open up a lot of options for easier access” (Yoga Teacher 4).

## Discussion

This qualitative study explored VA yoga teachers’ perspectives and experiences with implementing and delivering synchronous, group yoga-for-wellness classes via video-based telehealth. Yoga teachers interviewed for this study addressed a variety of needs for successful implementation and delivery of tele-yoga, which we grouped into 4 categories: (1) equipment and IT needs; (2) physical space; (3) accessibility and safety; and (4) administrative responsibilities and end-user support. The findings highlight VA yoga teachers’ main concerns and priorities regarding tele-yoga implementation and delivery, and strategies and resources they perceived as necessary for offering a safe, accessible, and effective experience for a clinical and medically complex population.

Findings from this study identified that yoga teachers required high quality audio-visual equipment to effectively deliver tele-yoga in a setting where health and safety are chief concerns. Teachers described situations where they made do with basic AV equipment, such as a laptop with a built-in camera, but also discussed ways in which their efforts to ensure safety and provide an effective experience were hindered by only having access to such basic AV equipment. While most sites were able to obtain recommended AV equipment (eg, Bluetooth headsets, large monitors/screens, and high-resolution webcams) shortly after onboarding to the study, others struggled due to site-specific challenges with purchasing new equipment. Additionally, teachers also reported issues with the telehealth platforms approved for delivery of tele-yoga and underlined the need for reliable internet connectivity. These findings are in accordance with a prior study conducted in VA examining the transition of CIH modalities to telehealth during the COVID-19 pandemic,^23^ indicating the need for prioritizing availability of high-quality network access to support virtual delivery of movement-based group classes. Interview participants also highlighted the importance of telehealth training, as many teachers in our study reported challenges with navigating VVC in a group format, instead of the one-one-one format with which they were more familiar as clinicians. Because VVC was not created with group classes in mind, the user interface often limited the ability to see all participants at the same time. It is important to note that VA has made interface updates to VVC since the conclusion of this study; however, limitations still exist for delivering group movement classes.

Findings from this study were also consistent with previous studies that highlight patient safety as a central concern for both teachers and yoga programs.^7,13,15-17,22-25,32^ A key challenge that was consistent with a prior study among a medically complex population in an online yoga setting^23^ was how to ensure a safe, accessible, and effective tele-yoga experience for participants when not every participant could be seen simultaneously by the teacher. While some sites limited participation to patients with clearance from a medical provider, safety protocols were necessary for falls prevention and other types of medical emergencies. Teachers also adapted by having participants move gently through different poses, by checking in with participants often during class, and by learning about their participants through chart review or an intake process. Yoga teachers described how they found opportunities to stretch their own capacity for adapting yoga to meet the needs of heterogenous, clinical populations they encountered in their classes, especially for older veterans and those with mobility limitations. Teachers also spoke eloquently about the need for sensitivity and training to make classes accessible to veterans with a history of trauma and for those managing PTSD and other mental health conditions.

The present study adds to the emergent literature on tele-yoga by highlighting the need for adequate physical space for teachers to deliver effective classes where participants are able to have a full-body view of the teacher.^15,17^ While a few teachers reported being able to occasionally teach yoga from home, most reported teaching from a VA healthcare facility. Those who taught from home discussed the importance of a well-lit, decluttered, distraction-free room for effective delivery. Those who taught from a VA healthcare facility often had to adapt their personal offices or multipurpose conference rooms for tele-yoga delivery. For some, teaching from these healthcare facility spaces presented challenges both for mounting needed AV equipment and for effectively demonstrating poses and movements in front of said AV equipment. While adapting different spaces for tele-yoga added to teachers’ competencies, it also added to their workload. Many described how advantageous it would be to have a dedicated room onsite for virtual CIH classes, and some mentioned how allowing yoga teachers to telework and teach yoga from home could remedy the spatial limitations they face when teaching onsite.

Our findings demonstrate that yoga teachers in healthcare settings must be flexible, proactive, and adaptable both to the unique needs of their patients and to the guidelines established by their employer. Although our interviewees were equipped with the knowledge, skills, and competencies required to teach yoga to veterans in VA, they were not necessarily trained to teach yoga in a synchronous, online format to a heterogenous patient population. This presented different challenges that yoga teachers had to manage and overcome, but it also presented unique opportunities for developing new competencies related to yoga instruction and technology utilization. The VA has developed an innovative yoga teacher training program that includes specialized training in trauma- and pain-informed yoga instruction and video-based telehealth delivery. The VA and other healthcare settings have an opportunity to leverage this model with healthcare providers trained in yoga both to increase yoga teacher workforces and to potentially reach medically complex populations that many community-based yoga studios and yoga in fitness centers are not typically equipped to serve. Teachers in this study sought to de-emphasize physically intense movement in favor of proprioception and body awareness, orientating participants towards a more meditative approach to yoga that integrates more breathwork, mindful movement, and meditation. This is an approach that can be translated to both within and outside the VA in other healthcare settings seeking to implement tele-yoga. Although most of the tele-yoga classes in this study focused on overall wellness and not condition-specific yoga therapy that would require greater specialization and training, the clinical background of most of the teachers in this study allowed them to serve the large number of patients with complex medical and mental health conditions seen in the VA setting. Future studies might examine the effectiveness of yoga teacher background and training (eg, yoga teacher or certified yoga therapist with or without dual clinical licensing) for effectiveness and implementation outcomes.

A finding that illustrated the importance of incorporating yoga teacher perspectives in our assessments of tele-yoga programs related to the amount of work actually involved in teaching tele-yoga in a healthcare setting. We found that VA yoga teachers not only take on additional responsibilities beyond teaching but must also consider many different factors as compared to yoga teachers in non-healthcare settings (eg, a privately-owned studio). Our results demonstrate that the workload associated with teaching and maintaining a telehealth-based yoga program can be high, especially when it is being delivered to groups instead of one-on-one. Yoga teachers in this study described less visible, “behind the scenes” responsibilities related to recruiting participants, evaluating referrals, emailing and meeting with veterans before and after classes, scheduling classes, creating and sending out weblinks, troubleshooting technology, and assisting veterans with their own technology needs; responsibilities that sometimes exceeded the number of hours they were assigned or contracted to work as part of the multi-site tele-yoga implementation study. Beyond professional development opportunities to expand skills to teach yoga in an online, synchronous format, yoga teachers need additional support in other areas to effectively deliver classes and enhance access for veteran participants. This includes, as mentioned above, adequate physical space (ie, well-lit and adequately sized rooms for positioning and demonstrating) for tele-yoga delivery, administrative support to lessen the burden of simultaneously teaching and running a tele-yoga program, and IT support both for teachers when they experience telehealth platform delivery issues and for participants when technology issues inevitably arise.

Although yoga teachers are critical for yoga delivery in healthcare settings, they often occupy positions at the bottom of the healthcare professional hierarchy unless they are also licensed clinicians. This creates challenges both in terms of hiring experienced yoga teachers with the ability to adapt and tailor classes in real time and also in terms of the expectations and demands that can be placed on yoga teachers as it relates to administrative responsibilities that often accompany teaching yoga in a healthcare setting, in all its forms: in-person, virtual only, and hybrid. Given the benefits of implementing tele-yoga within healthcare systems, which includes above all reaching patients that might otherwise not have access to yoga in their communities or for health reasons, it is vital that the delivery and workload issues highlighted here be addressed.

To successfully develop, staff, implement, deliver, and expand virtual group yoga offerings in healthcare settings, we need to continue developing our understanding of the resources (eg, equipment, trainings, space, supplies, personnel) that yoga teachers need to deliver safe, accessible, and effective tele-yoga classes, and it’s crucial that we hear from yoga teachers themselves about what is needed.^23,32^ Given their role in tele-yoga implementation and delivery, VA yoga teachers have unique insight on what is required to successfully launch and sustain tele-yoga in one-on-one or group formats. At the present moment, there are too few studies examining yoga teachers’ experiences delivering yoga to clinical and medical complex populations (see Ellis et al 2022 and Haynes et al 2022 for 2 exceptions).^23,32^ Healthcare organizations that seek to implement or expand virtual yoga offerings for different clinical conditions and general wellbeing would greatly benefit from learning about the strategies and techniques VA yoga teachers have used to address specific populations and clinical conditions in virtual classes, particularly in heterogenous group classes. VA yoga teachers also have vital information to share as it relates to balancing different clinical responsibilities (eg, teaching and documenting yoga encounters and seeing patients in a different clinical capacity), all the while navigating the unique delivery issues that arise in large healthcare settings like VA; information that can yield practical implications for how to build and sustain a tele-yoga program and workforce that is flexible, adaptable, and resilient.

### Limitations

This study of the experiences of yoga teachers delivering tele-yoga classes across several VA sites has certain limitations. First, we interviewed teachers in different stages of the implementation process, so perspectives and experiences could have changed across the course of the implementation period as sites increased the program infrastructure and as teachers themselves became more familiar with VA processes and systems. Secondly, both VA sites and yoga teachers themselves varied in their experience specifically teaching online; some had pivoted rapidly to offering yoga online because of the COVID-19 pandemic, while others started teaching yoga online as part of the tele-yoga study and were learning as they worked. This variation in online teaching experience could have had some effect on teachers’ perspectives. However, a strength of this study lies in the unique insights shared by yoga teachers with dual clinical licensing and 3 plus years of experience working for VA. Finally, this paper presents the perspectives of only yoga teachers and does not include the experiences of other VA staff involved in the implementation of the tele-yoga program or the views of Veteran participants in the classes. However, future publications will address these perspectives for a more comprehensive picture of VA tele-yoga for wellness delivery.

### Implications for Policy and Practice

This evaluation of a multi-site tele-yoga implementation study shows one area where telemedicine advancements made during the COVID-19 pandemic have proven successful and should continue to be supported. Labor mapping, importantly, provided one avenue to establish and increase the yoga teacher workforce across the VA health care systems participating in this study. Further growth of this program could develop upon the labor mapping approach in addition to drawing from current VA internal yoga teacher trainings. Advancements are being made to VVC to accommodate movement-based group classes, creating new opportunities for enhancing access to yoga. Demand for yoga classes is high in VA. Yoga teachers stated that Veterans wanted more classes available across days and times as well as more diverse yoga offerings. Tele-yoga programs would benefit from support at multiple levels across healthcare systems including administrators with funding allocation authority. Allocating additional resources for teachers and veterans could enhance access, help to make tele-yoga programs safer, and sustain a program that is popular with veterans. Investing more resources in the tele-yoga programs would help to make yoga more widely available to rural and other Veterans in need and could help to improve health outcomes and engagement in healthy living.

## Conclusion

This qualitative study among VA yoga teachers identified key considerations for implementation and delivery of tele-yoga within healthcare settings including equipment and IT needs, adequate physical space, accessibility and safety, and administrative responsibilities. As the evidence base for yoga for health conditions and overall health and wellbeing expands, insights from VA tele-yoga teachers can inform best practices and recommendations as tele-yoga programs expand within VA and into other healthcare systems.
